# Catalogues and Annual Circulars

**Published:** 1848-02

**Authors:** 


### CATALOGUES AND ANNUAL CIRCULARS.

Catalogue of the officers and Students of the Medical Department of Hampden Sidney College, in Richmond, Virginia. Session of 1846–7.
Number of the Class, 67. Number of Graduates, 17.

Annual Circular and Catalogue of the Medical Department of the University of Buffalo.
Number of the Class, 67. Number of Graduates, 18.

Fifth Annual Announcement for 1847–48, and Catalogue of the Rush Medical College, Chicago, Illinois.
Number of the Class, 70. Number of Graduates, 17.

